# Contact Load Measurement and Validation for Tapered Rollers in Wind Turbine Main Bearing

**DOI:** 10.3390/s25154726

**Published:** 2025-07-31

**Authors:** Zhenggang Guo, Jingqi Yu, Wanxiu Hao, Yuming Niu

**Affiliations:** School of Mechanical Engineering, Dalian University of Technology of China, Dalian 116024, China

**Keywords:** wind turbine main bearing, tapered roller bearing, contact load, strain measurement, phase compensation, sensor design, bracket design

## Abstract

**Highlights:**

**What are the main findings?**

- An analysis was conducted on the issue of roller inner bore measurement point sensitivity, and a bracket structure for strain measurement was proposed.
- A strain–phase–load calculation method considering the roller’s rotation phase was proposed.

**What is the implication of the main finding?**

- This structure can effectively capture strain signals while ensuring the overall structural stiffness of the roller after drilling.
- This method enables real-time acquisition of contact load during roller rotation, which is of great significance for improving bearing operational reliability.

**Abstract:**

Addressing the need for contact load detection in wind turbine main bearings during service, a roller contact load measurement method is proposed. An analytical model characterizes the contact load-to-inner bore strain mapping relationship. To overcome the inherent low sensitivity of direct bore strain measurement, bore-to-measurement-point sensitivity analysis was optimized. Multiple structurally optimized sensor brackets were designed to enhance strain measurement sensitivity, and their performance was comparatively evaluated via simulation. To mitigate sensitivity fluctuations caused by roller rotation phase variations, a strain–phase–load calculation method incorporating real-time phase compensation was developed and verified through simulation analysis. A dedicated roller contact load testing system was constructed and experimental validation was conducted. Results demonstrate 95% accuracy in contact load acquisition. This method accurately obtains roller contact loads in wind turbine main bearings, proving crucial for studying bearing mechanical behavior, predicting fatigue life, optimizing structural design, and enhancing reliability.

## 1. Introduction

As a core component of many offshore wind turbine generator systems, the main bearing typically employs a large single-row tapered roller bearing to meet the demands of its complex operating conditions. Within the bearing’s internal structure, the contact area between the tapered rollers and the raceways constitutes a critical region characterized by highly concentrated stress and the highest risk of failure. During bearing operation, significant contact loads are generated between the rollers and the inner and outer rings [1]. The magnitude of these loads profoundly influences the bearing’s overall performance and service life [2,3]. Consequently, accurately acquiring the contact loads at the roller–raceway interface is of paramount importance for in-depth studies of bearing mechanical behavior, fatigue life prediction, structural design optimization, and enhancing overall reliability [4,5].

Due to the difficulty of directly measuring roller contact load, major international wind turbine bearing manufacturers such as SKF and FAG have developed methods to measure internal strain within the rollers to indirectly acquire the contact load [6,7,8,9,10]. Within this technical framework, investigating the mapping relationship between contact load and bore strain, as well as the sensitivity of measurement points, becomes two critical issues for accurately obtaining the roller contact load in wind turbine generators. Regarding research on the roller load and strain, Chen et al. [11] have comprehensively elucidated the analytical methods for the elastic approach in line contact problems, solving and comparatively analyzing them using different methods. Tong and Hong [12] analyzed the effect of angular misalignment on the operating torque of tapered roller bearings and proposed a method for estimating the contact load on a single tapered roller. Kabus [13] employed the elastic half-space theory to derive and calculate the contact stress on tapered roller surfaces and subsequently calculated the fatigue life of tapered roller bearings. Yang et al. [14] analyzed the contact between the roller and raceway based on the finite length contact theory and determined the maximum contact stress of the bearing via an iterative solution. Warda [15] utilized a three-dimensional elastic body contact method to compute roller contact stress and verified the results via ANSYS simulation. Wei et al. [16] applied VB programming to calculate the load distribution in tapered roller bearings and simulated the contact load on tapered rollers under various operating conditions. Chen et al. [17] derived the equilibrium equations for a single tapered roller bearing under load, established a mathematical model for overall contact analysis, and solved the model using MATLAB programming. Ju et al. [18] focused on tapered roller bearings, employing central control and Hertz contact theory to study the contact stress on their surfaces. Tie et al. [19] calculated the contact load of tapered roller bearings based on Hertz contact theory and achieved consistency between Romax Designer simulation results and theoretical calculations. Li et al. [20] used an equivalent substitution approach to establish three mathematical models for calculating roller contact load and deformation. Their proposed new method enables rapid calculation of roller contact deformation at different time points. D. V. Raju [21] provided theoretical methods for calculating roller contact stress under different operating conditions, enabling accurate prediction. The accuracy of the method was verified through finite element analysis using ABAQUS. Cheng et al. [22] investigated the load distribution within tapered roller bearings using numerical analysis and simulated the contact stress state using Abaqus software, serving as the basis for bearing design and life assessment. To address the computational efficiency of the finite element method and resolve non-convergence issues, Chen et al. [23] proposed a finite element contact behavior calculation method using a spring substitution technique, effectively obtaining the contact stress between rollers and raceways. Wang et al. [24] established a calculation model for roller–raceway contact stress and contact load based on Hertz contact theory and the roller slice theory. Luo et al. [25], based on the fundamental equations of elastic contact problems and considering curvature variations in tapered roller cross-sections, proposed a simplified calculation method and procedure for tapered roller contact stress, demonstrating its validity through programmed examples. Li et al. [26] conducted a global analysis of tapered roller bearings in wind turbines using PERMAS, studying the influence of axial clearance on roller load distribution. Liu et al. [27] investigated the influence of mesh precision on contact calculations in finite element analysis, providing guidance for the precise finite element modeling and analysis of roller bearings. Wei et al. [28] applied the finite element method to study the internal loads and stresses in tapered roller bearings during loading.

Although the aforementioned research has laid the foundation for establishing the relationship between contact load and roller strain, it lacks an in-depth investigation specifically targeting wind turbine main bearings. Furthermore, research on the mapping relationship for bore strain is notably absent. Regarding studies on strain measurement sensitivity, Shi et al. [29] designed a structure for high-precision acquisition of minute strains. Characterized by a dumbbell-shaped form (thick ends and a thin middle section), this structure functions to transfer and amplify small strains. Li et al. [30] combined the lever amplification principle to design an amplification structure capable of increasing the axial strain of fiber Bragg gratings (FBG), thereby enhancing sensor response sensitivity. Zheng et al. [31] effectively captured the dynamic variations of strain using a rhombic displacement amplification mechanism. Additionally, strain measurement structures such as variable-section ring shapes [32], among others, have been employed, all contributing to improved strain measurement sensitivity. Wang et al. [33] arranged multiple load sensors along the roller’s radial direction. While increasing the number of sensors mitigated the influence of roller rotation on contact load measurement, this approach also increased system complexity and elevated circuit power consumption. FAG Corporation [34] has developed an externally mounted smart bearing that integrates multiple sensors such as temperature, rotational speed, and vibration. The sensors are installed within the sealing groove of the bearing’s outer ring, which increases the structural complexity of the bearing. Based on the existing literature, the research mentioned above has yielded initial achievements in acquiring bearing contact loads, reaching the stage of functional realization. However, research concerning strain measurement sensitivity remains in its nascent stage, with the underlying theories and technical solutions requiring further significant development.

To address the aforementioned issues, this study constructs a contact load–strain analytical model, revealing the mapping relationship between contact load and bearing bore strain. Through simulation analysis, the optimal layout scheme for sensor brackets and the distribution regions of high-sensitivity measurement points are determined under given bore strain conditions. Sensor brackets are strain amplification structures installed within the roller bore, serving as a bridge between strain gauges and the roller. They transmit and amplify deformation of the roller bore. Mounting sensors on these brackets enhances the sensitivity of strain signal measurements. Sensor brackets are strain amplification structures installed within the roller bore, serving as a bridge between strain gauges and the roller. They transmit and amplify deformation of the roller bore. Mounting sensors on these brackets enhances the sensitivity of strain signal measurements. Based on this, a strain–phase–load calculation method incorporating roller rotation phase factors is further established, enabling the accurate extraction of contact load. This method was validated through experimental testing. Both theoretical and experimental results demonstrate that the proposed strain–phase–load calculation method can acquire the contact load on main bearing rollers in wind turbine generator systems in real time and with high precision. This, thereby, provides effective technical support for in-depth research on bearing mechanical characteristics, fatigue life prediction, and structural design optimization.

## 2. Contact Load–Bore Strain Mapping

Due to the extremely high contact stress experienced at the tapered roller–raceway contact interface, it is impractical to directly deploy sensors on the contact surface for load measurement. Consequently, in engineering practice, machining a bore within the roller can be adopted. Contact load is then indirectly acquired by measuring the strain on the inner wall of this bore. Therefore, an in-depth investigation into the quantitative mapping relationship between the strain on the roller bore wall and the contact load is essential. Furthermore, tapered roller bearings possess inherently complex geometric parameters, leading to a particularly complex stress distribution state under load [35]. To address these challenges, this chapter focuses on the mechanical analysis of the deformation behavior of loaded rollers. A relationship model between the surface strain of a solid roller (without a bore) and the contact load is established. Building upon this model, analytical calculation formulas for the bore surface strain of a hollowed tapered roller and its relationship to the contact load are derived. This provides a solid theoretical foundation for acquiring contact load through bore strain measurement.

**Figure 1:**
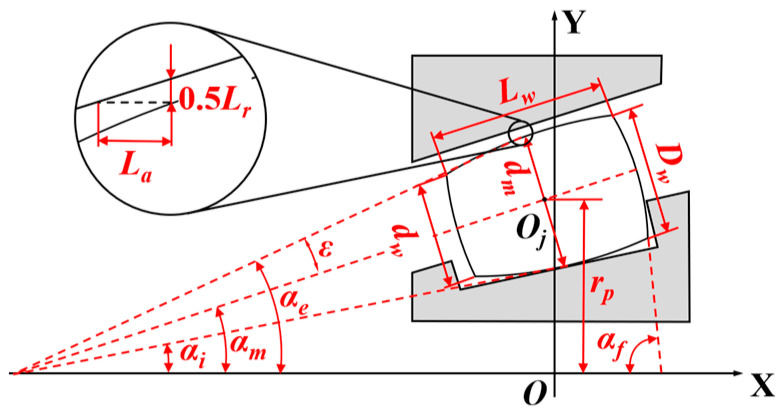
The tapered roller bearing model.

To clarify the geometric relationships involved in the subsequent analysis, the key geometric parameters of the tapered roller bearing are first defined, as illustrated in Figure 1. Point O is designated as the bearing center point, and point O*_j_* is designated as the center point of an individual roller. The specific meanings of the parameters are as follows:

- *d_w_*: Roller small end diameter;
- *D_w_*: Roller large end diameter;
- *d_m_*: Roller mean diameter (diameter at the axial midpoint);
- *r_p_*: Bearing pitch circle radius;
- *L_w_*: Roller effective contact length;
- *L_a_*: Bearing axial clearance;
- *L_r_*: Bearing radial clearance;
- *α_i_*: Contact angle between roller and inner ring raceway;
- *α_e_*: Contact angle between roller and outer ring raceway;
- *α_m_*: Roller mean contact angle;
- *ε*: Roller semi-cone angle;
- *α_f_*: Roller rib face angle.

Although the classical Hertz line contact theory provides the theoretical basis for calculating the contact load between rollers and raceways, it exhibits significant limitations when applied to tapered rollers and fails to yield an analytical solution formula for contact deformation [17,36]. Consequently, for non-spherical roller bearings (such as tapered roller bearings), analysis typically relies on the empirical formula proposed by Palmgren [37]. This formula establishes a quantitative relationship between the contact load and contact deformation for a given roller. The specific expression is as follows:

(1) $$Q=K\delta^{n}$$

(2) $$K=8.06\times 10^{4}L^{8/9}$$

The specific meanings of the parameters in the formula are as follows:

- *Q*: Contact load;
- *δ*: Contact deformation;
- *K*: Load–deformation constant;
- *L*: Contact length between the roller and raceway;
- *n*: Load–deformation exponent (for a given tapered roller, *n* = 10/9).

**Figure 2:**
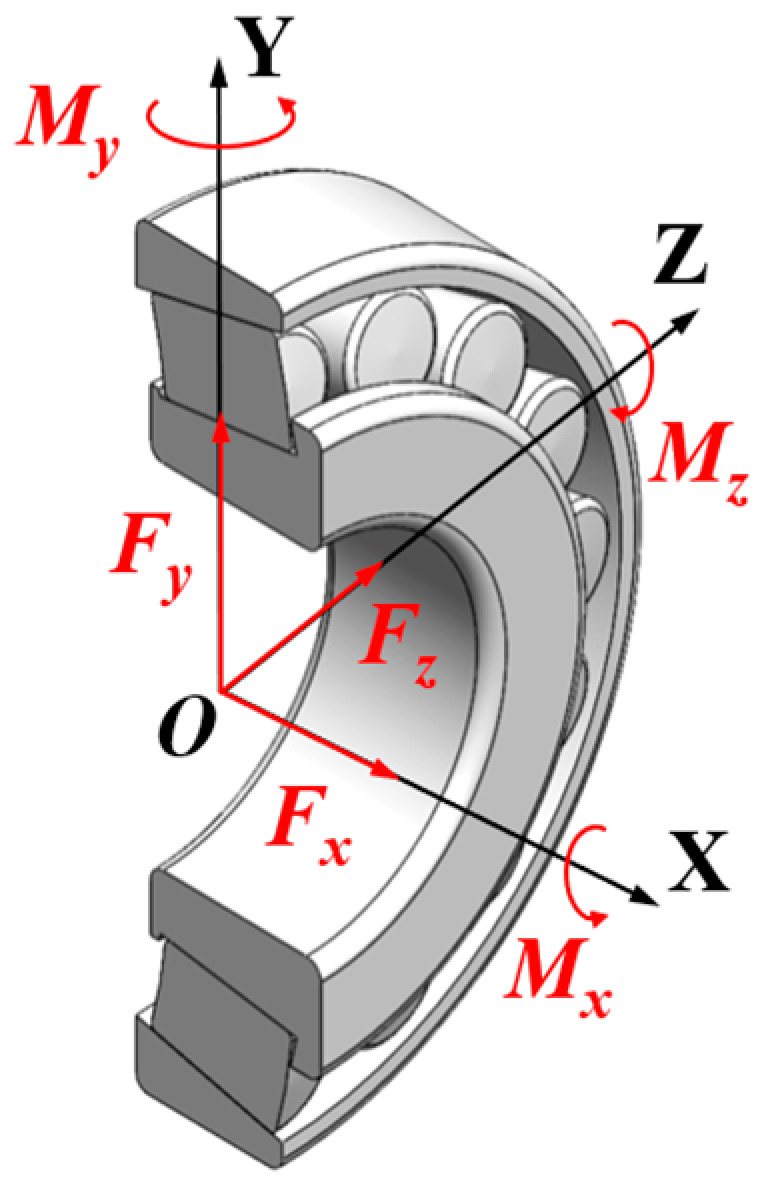
The global coordinate system.

To analyze the contact load on the roller, a global coordinate system is established with the bearing center point O as the origin, as shown in Figure 2. The *X*-axis is defined along the bearing axial direction. Within this coordinate system, the external force acting on the bearing can be resolved into three components along the coordinate axes: *F_x_*, *F_y_*, *F_z_*. Simultaneously, the external moment acting on the bearing can be resolved into components about the three coordinate axes: *M_x_*, *M_y_*, *M_z_*.

**Figure 3:**
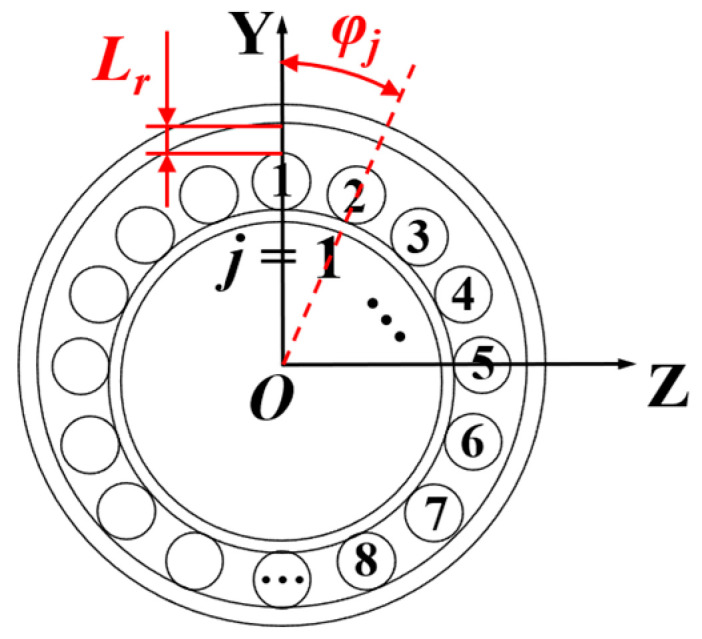
The absolute position angle and radial clearance.

The symbol *j* is used to denote the roller number. The positive direction of the global coordinate system’s *Y*-axis is defined to point toward Roller No. 1. Subsequently, the roller number *j* increases sequentially in a clockwise direction around the *X*-axis, as illustrated in Figure 3. The absolute position angle, denoted as *φ_j_*, is defined for the *j*-th roller. This angle represents the circumferential angle of the roller’s center relative to the origin of the bearing’s global XYZ coordinate system. A specific mathematical relationship exists between the roller’s absolute position angle *φ_j_*, its number *j*, and the total number of rollers *z*. This relationship can be expressed as:

(3) $$\varphi_{j}=\frac{2\pi }{z}\cdot j$$

**Figure 4:**
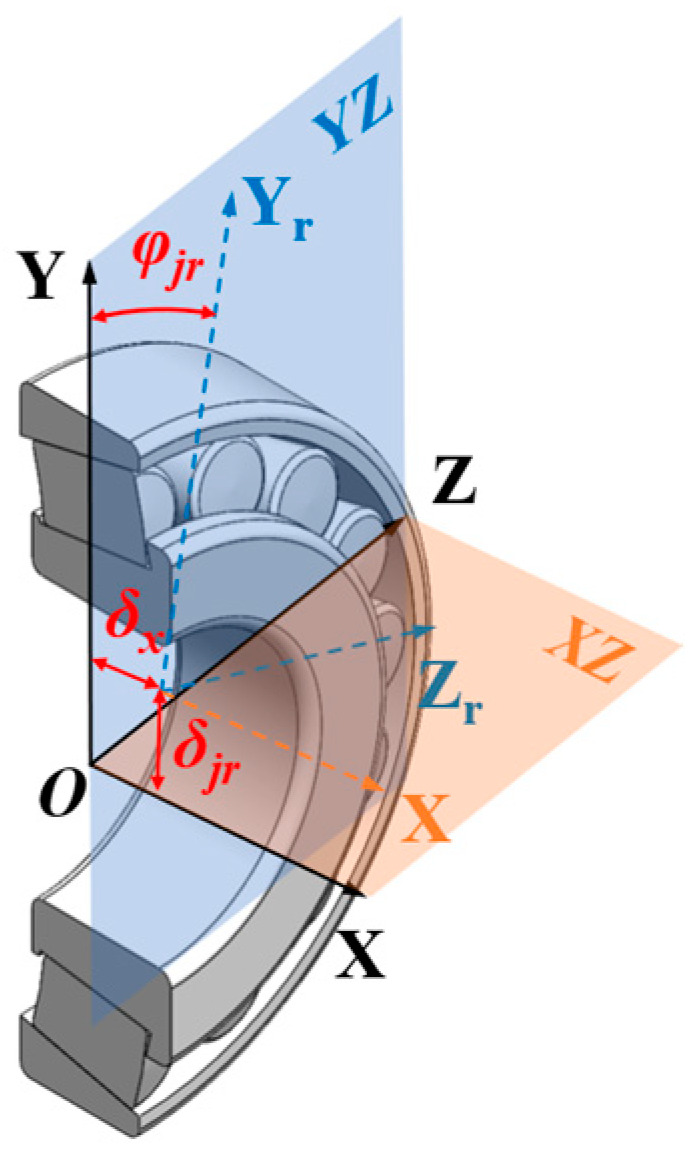
Rotation of the global coordinate system XYZ.

When the direction of the resultant force of the external load does not align with the *Y*-axis of the global coordinate system, it is necessary to decompose the force. To simplify subsequent mechanical analysis, it is assumed that the roller is rigidly connected to the inner ring of the bearing. Under this assumption, the original global coordinate system XYZ is rotated by a certain angle around its *X*-axis to establish a new coordinate system XY_r_Z_r_. The spatial relationship after rotation is illustrated in Figure 4. Under external loading, both the roller and inner ring will undergo displacement, with a radial offset of *δ_jr_* and an axial offset of *δ_x_*. Simultaneously, the rotation angle *φ_jr_* is defined as the relative position angle of the *j*-th roller, which is specifically characterized as follows:

(4) $$\varphi_{jr}=\arctan\left(F_{z}/F_{y}\right)$$

**Figure 5:**
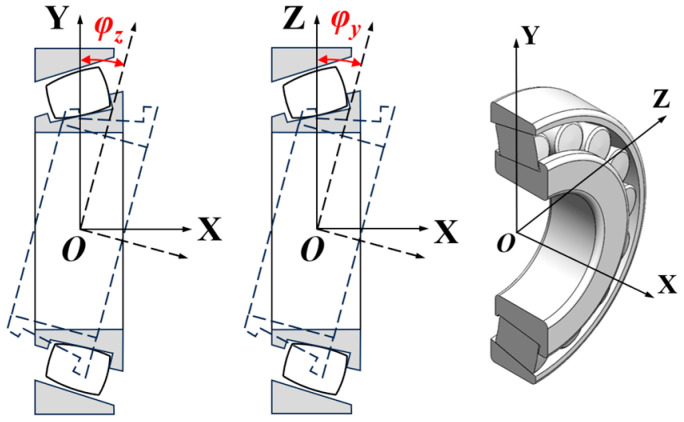
Inner ring deflection angle of the bearing.

When the bearing is subjected to both axial and radial loads simultaneously, it is assumed that the outer ring remains stationary. Under these conditions, the inner ring of the bearing will deflect relative to the outer ring about the *Y*-axis and *Z*-axis of the global coordinate system. These deflection angles are denoted as *φ_y_* and *φ_z_*, respectively, as illustrated in Figure 5.

**Figure 6:**
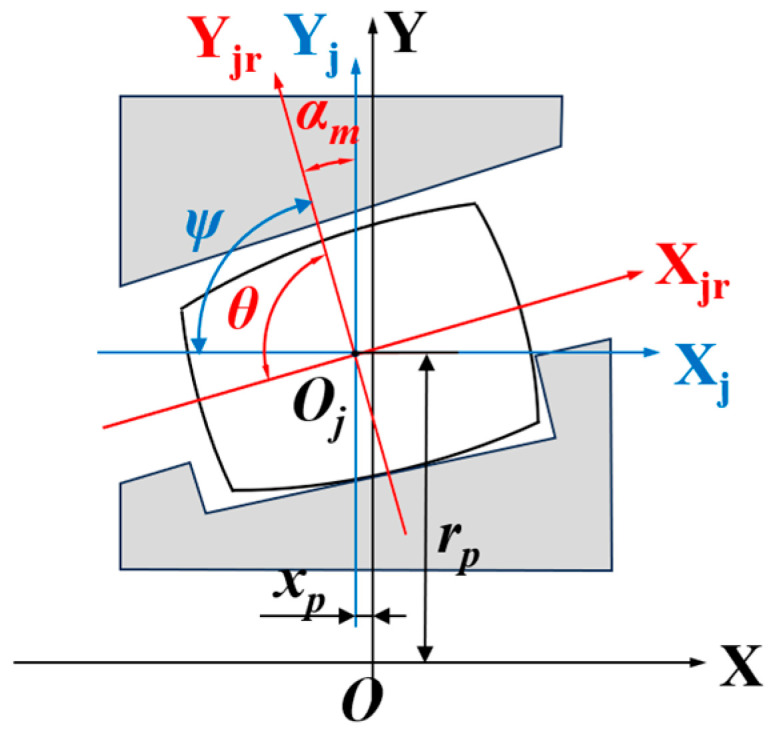
The roller coordinate system *X_j_Y_j_θ* and roller rotating coordinate system *X_jr_Y_jr_ψ*.

With the centroid *O_j_* of the *j*-th roller as the origin, a local roller coordinate system *X_j_Y_j_θ* is established. Subsequently, this coordinate system is rotated by an angle *α_m_* about its origin to obtain a new roller coordinate system *X_jr_Y_jr_ψ*, as shown in Figure 6. Given that the axial offset of the roller centroid *O_j_* relative to the bearing center *O* is *x_p_*, the coordinates of the roller coordinate system origin in the global coordinate system are (*x_p_*, *r_p_*). To analyze the force-induced deformation of the roller, the displacement offset vector *δ* generated by the load in the global coordinate system XYZ must be transformed into the roller’s own coordinate system *X_j_Y_j_θ*, thereby deriving the relationship between them:

(5) $$\left\{\begin{array}{c} \delta_{X_{j}}=\delta_{x}+r_{p}\varphi_{y}\sin\varphi_{j}+r_{p}\varphi_{z}\cos\varphi_{j}-L_{a}\\ \delta_{Y_{j}}=\delta_{jr}\cos\left(\varphi_{j}-\varphi_{jr}\right)-x_{p}\varphi_{y}\sin\varphi_{j}-x_{p}\varphi_{z}\cos\varphi_{j}-L_{r}/2\\ \theta =\varphi_{y}\sin\varphi_{j}+\varphi_{z}\cos\varphi_{j} \end{array}\right.$$

To simplify calculations, the aforementioned relationship is decomposed into matrix form:

(6) D=Tδ−L

where vector *D* represents the offset vector in the roller coordinate system *X_j_Y_j_θ*, *T* is the transformation matrix, and *L* denotes the bearing clearance matrix. These can be expressed as:

(7) $$D={\left[\begin{array}{ccc} \delta_{X_{j}} & \delta_{Y_{j}} & \theta \end{array}\right]}^{T}$$

(8) $$T=\left[\begin{array}{cccc} 1 & 0 & r_{p}\sin\varphi_{j} & r_{p}\cos\varphi_{j}\\ 0 & \cos\left(\varphi_{j}-\varphi_{jr}\right) & -x_{p}\sin\varphi_{j} & -x_{p}\cos\varphi_{j}\\ 0 & 0 & \sin\varphi_{j} & \cos\varphi_{j} \end{array}\right]$$

(9) $$\delta ={\left[\begin{array}{cccc} \delta_{x} & \delta_{jr} & \varphi_{y} & \varphi_{z} \end{array}\right]}^{T}$$

(10) $$L={\left[\begin{array}{ccc} L_{a} & L_{r}/2 & 0 \end{array}\right]}^{T}$$

Based on the relationship between coordinate systems *X_j_Y_j_θ* and *X_jr_Y_jr_ψ* shown in Figure 6, we further derive:

(11) U=RD

where vector *U* represents the offset vector in the rotated roller coordinate system *X_jr_Y_jr_ψ*, and *R* is the rotation matrix. These can be expressed as:

(12) $$U={\left[\begin{array}{ccc} \delta_{X_{jr}} & \delta_{Y_{jr}} & \psi \end{array}\right]}^{T}$$

(13) $$R=\left[\begin{array}{ccc} \cos\alpha_{m} & \sin\alpha_{m} & 0\\ -\sin\alpha_{m} & \cos\alpha_{m} & 0\\ 0 & 0 & 1 \end{array}\right]$$

**Figure 7:**
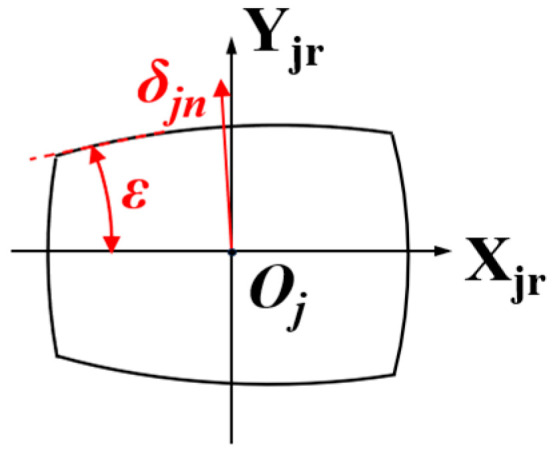
Normal offset of the tapered roller.

When contacting the outer raceway, the normal contact deformation offset *δ_jn_* of the tapered roller is illustrated in Figure 7. This offset is derived from the displacements along the *X_jr_*-axis and *Y_jr_*-axis combined with the semi-cone angle *ε*:

(14) $$\delta_{jn}=\delta_{X_{jr}}\sin\varepsilon +\delta_{Y_{jr}}\cos\varepsilon$$

**Figure 8:**
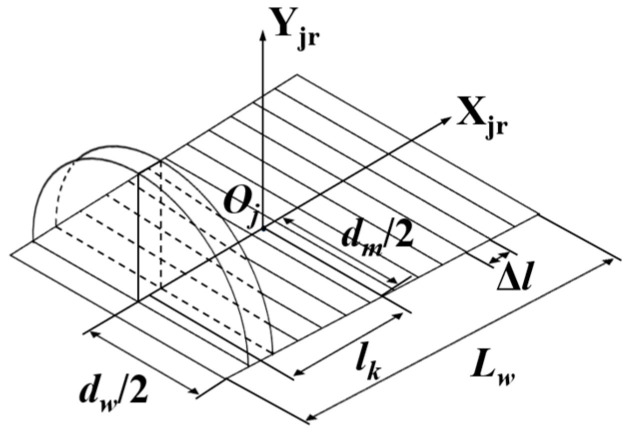
Roller slice division.

The tapered roller is discretized along its axis into *n* slices of uniform thickness Δ*l*, where each slice is approximated as a constant-diameter cylinder, as shown in Figure 8. The position *l_k_* of the *k*-th slice is calculated as follows:

(15) $$l_{k}=\left(k-1/2\right)\Delta l$$

When an ideal cylindrical roller contacts the raceway, its contact stress exhibits a uniform rectangular distribution along the contact line. However, due to the convergent geometry of tapered rollers, stress concentration occurs at both ends of the contact zone. This results in significantly higher stress values in these localized regions than predicted by classical Hertz line contact theory [38]. To improve the stress distribution and promote a more uniform profile, logarithmic profile modification is applied to the tapered roller contour. The modification depth along the roller axis is given by:

(16) $$h=4.5\times 10^{-4}d_{m}\ln\frac{1}{1-{\left(\frac{2l_{k}}{L_{w}}\right)}^{2}}$$

where *d_m_* denotes the mean diameter of the profiled tapered roller, and *L_w_* represents the effective contact length of the roller.

**Figure 9:**
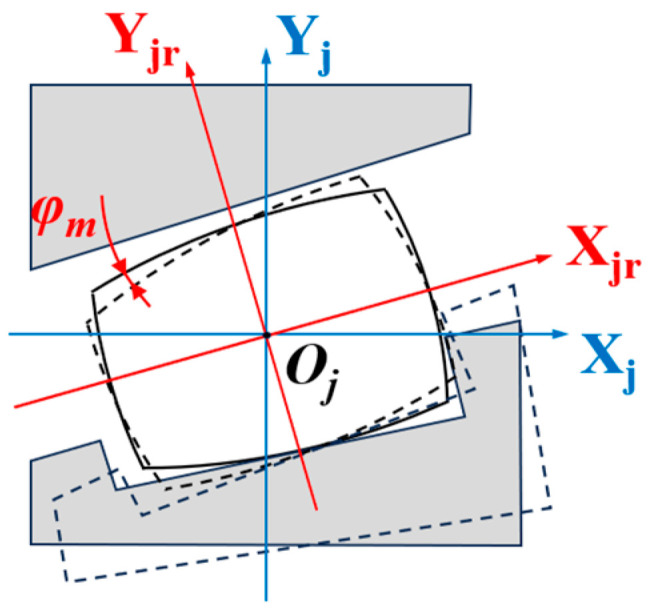
Roller misalignment angle.

Consequently, when calculating the contact deformation of each discrete slice, the influence of roller profiling must be considered. Furthermore, due to the misalignment angle *φ_m_* inducing additional compressive (or separative) deformation at the slice locations, as illustrated in Figure 9, a corrective adjustment for this effect is required. Synthesizing the profiling influence and misalignment correction, the equivalent normal contact deformation for each slice is derived as follows:

(17) $$\delta_{jnk}=\delta_{jn}+h_{m}-h$$

(18) $$h_{m}=\left(-l_{k}\right)\varphi_{m}$$

where *h_m_* denotes the correction term for the slice’s additional deformation induced by angular misalignment, and *l_k_* represents the position coordinate of the *k*-th slice along the *X_jr_*-axis, with the roller center (*l_k_* = 0) as the origin and the positive direction defined toward the roller’s large end.

Deformation response under misalignment:

1. Small-end compression (*φ_m_* > 0)
  Small-end slices: Increased deformation *h_m_* > 0
  Large-end slices: Reduced deformation *h_m_* < 0 (potential separation)
2. Large-end compression (*φ_m_* < 0)
  Small-end slices: Reduced deformation *h_m_* < 0
  Large-end slices: Increased deformation *h_m_* > 0

Based on Equations (1) and (2), the contact load *q_jnk_* on each slice can be calculated using the contact deformation *δ_jnk_* of that slice. The roller’s total contact load *Q_jn_* is obtained by summing the contact loads of all slices. Crucially, when the contact deformation *δ_jnk_* computed via Equation (17) yields a negative value, it indicates loss of contact between the slice and the outer raceway. Consequently, for slices where *δ_jnk_* < 0, the corresponding contact load *q_jnk_* must be set to zero in the summation for *Q_jn_*. The final expression for the roller contact load accounting for separation states is given by:

(19) $$Q_{jnk}=\sum_{k=1}^{n}q_{jnk}$$

(20) $$q_{jnk}=\left\{\begin{array}{c} K\delta_{jnk}^{10/9},\;\delta_{jnk}>0\\ 0,\;\delta_{jnk}\leq 0 \end{array}\right.$$

Considering the case of a hollow tapered roller, the contact deformation on the inner wall *δ_hjnk_* is related to the outer surface contact deformation *δ_jnk_* by:

(21) $$\delta_{jnk}=K_{h}\delta_{hjnk}$$

where *K_h_* denotes the conversion coefficient. The relationship between strain and deformation on the inner wall is given by:

(22) $$\delta_{hjnk}=K_{\varepsilon }\varepsilon$$

Synthesizing Equations (20)–(22), the contact load on the slice is derived as:

(23) $$q_{jnk}=\left\{\begin{array}{c} K_{q}\varepsilon ,\;\delta_{jnk}>0\\ 0,\;\delta_{jnk}\leq 0 \end{array}\right.$$

(24) $$K_{q}=K{\left(K_{h}K_{\varepsilon }\right)}^{10/9}$$

## 3. Roller Bore Measurement Point Sensitivity Analysis and Optimization

Given the extraordinarily high structural stiffness of tapered rollers, the elastic deformation induced under load is minimal in magnitude. If strain on the inner bore wall were measured directly to characterize load variations, the strain signal variation would be too small for direct sensor detection. Therefore, a dedicated sensor bracket must be integrated between the bore and measurement points to amplify strain measurement sensitivity. This chapter presents the structural design of roller bore sensor bracket and evaluates the technical feasibility of alternative configurations.

### 3.1. Sensor Bracket Design

**Figure 10:**
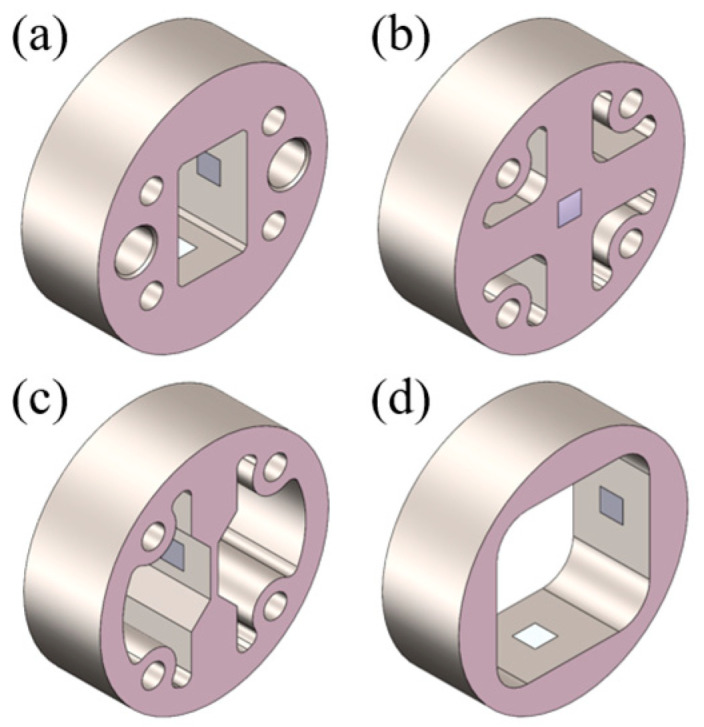
Four measurement schemes: (**a**) Rectangular measuring bracket; (**b**) Cross-shaped bracket; (**c**) Single-beam bracket; (**d**) Annular bracket.

**Figure 11:**
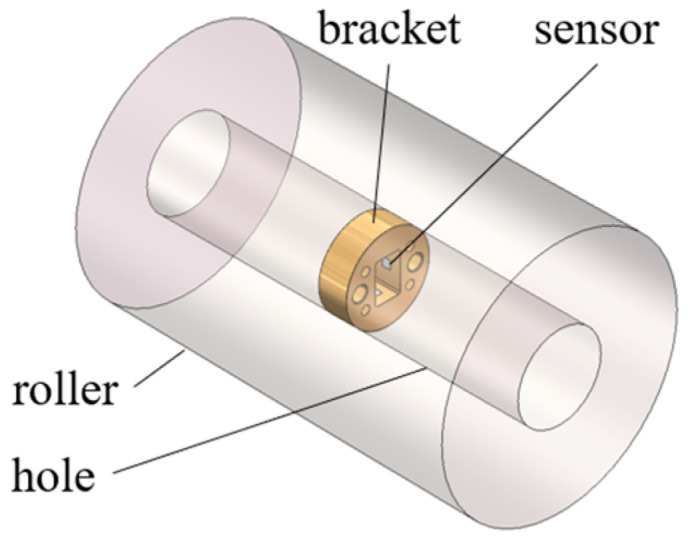
Bracket and roller assembly.

To meet the installation requirements for sensors in roller bore, four specific bracket measurement schemes are proposed, with their configurations illustrated in Figure 10. In Figure 10a–d, sensor brackets are positioned at the center of the roller bore via interference fits. Strain measurements are taken within designated instrumentation zones on these brackets, as detailed in Figure 11. This approach magnifies the roller’s structural deformation and facilitates strain acquisition. The mechanism transmits the roller’s minute deformations to the bracket’s measurement zones while providing amplification, thus enabling more effective collection and measurement of strain signals.

**Figure 12:**
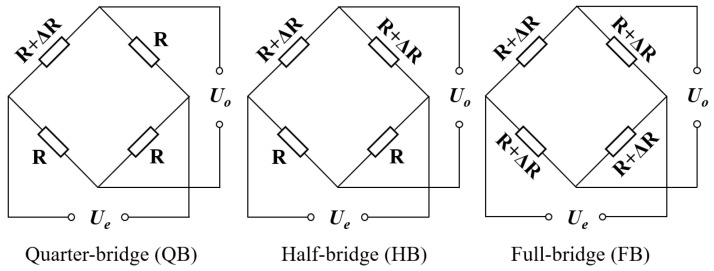
DC bridge configuration.

The connection configuration of the DC bridge is illustrated in Figure 12, where the bridge arm resistance is denoted as *R*, the resulting resistance variation as Δ*R*, the excitation voltage supplied to the bridge as *U_e_*, and the output voltage measured across the bridge as *U_o_*.

**Figure 13:**
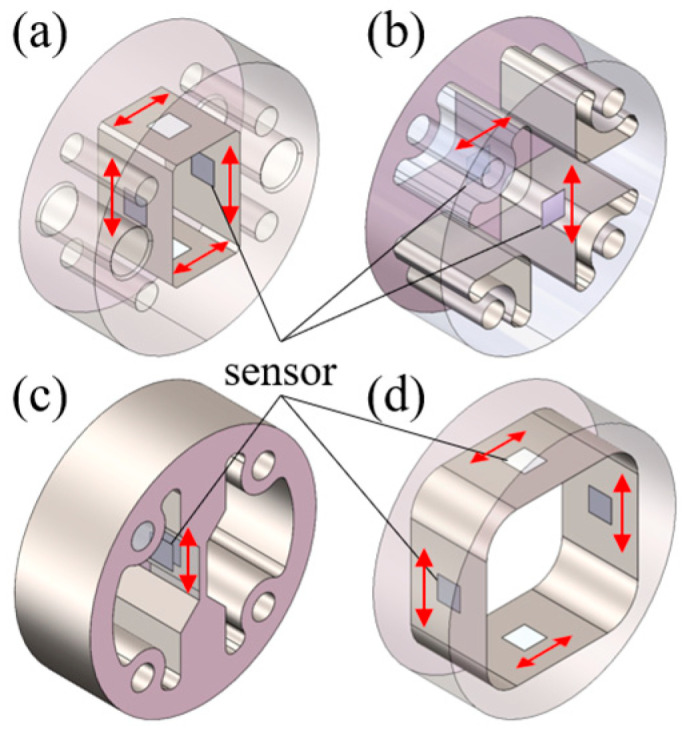
Strain measurement directions on the bracket: (**a**) Rectangular measuring bracket; (**b**) Cross-shaped bracket; (**c**) Single-beam bracket; (**d**) Annular bracket.

For the bracket in Figure 10a, strain gauges are mounted on the inner surface of the oblong hole. The measurement orientation of the gauges is shown in Figure 13a, with a full-bridge (FB) circuit configuration. This bridge configuration enhances the sensitivity of strain measurements. Several peripheral circular holes are designed for PCB positioning and interconnection. The structural asymmetry complicates the mapping relationship between strain and load. For the bracket in Figure 10b, strain gauges are mounted on both front and back surfaces at the central region. The gauges are orthogonally oriented, as depicted in Figure 13b, configured in a half-bridge (HB) circuit. This structure offers modest improvement in strain measurement sensitivity but significantly facilitates the mapping relationship between strain and load. For the bracket in Figure 10c, leveraging the high-strain sensitivity of thin-plate structures, strain gauges are attached to both sides of the flexure plate (see Figure 13c) in a quarter-bridge (QB) circuit. For the bracket in Figure 10d, strain gauges are installed on the inner surface of the rectangular hole (Figure 13d), employing a full-bridge (FB) circuit configuration. This structure integrates the full-bridge measurement capability of Bracket a with the symmetric design of Bracket b, thereby enhancing strain measurement sensitivity while simultaneously simplifying the strain–load mapping relationship.

Bearings generate heat during operation while being subjected to varying ambient conditions. These factors collectively influence bearing temperature, significantly impacting strain measurements using DC bridges. Consequently, temperature compensation must be incorporated into the bridge configuration. For full-bridge (FB) setups (Figure 13a,d), temperature compensation is inherently achieved through the circuit’s symmetry and subtractive properties. For half-bridge (HB) (Figure 13b) and quarter-bridge (QB) (Figure 13c) configurations, temperature compensation is implemented by connecting the inactive bridge arms to the bracket via thermal grease, alongside the active measuring arms, with additional potting applied to ensure reliable connections.

### 3.2. Structural Case Study of Hollow Tapered Rollers

This study focuses on the main bearing (Model: FL-306/1850X3/HCEYAD1, Wafangdian Bearing Group Corp., Ltd., Dalian, China) of a multi-megawatt wind turbine produced by a leading manufacturer. Geometric models of its tapered rollers, the inner ring, and the outer ring were established, with key structural dimensions summarized in Table 1. To reduce computational complexity, minor geometric features with negligible impact on analytical results, such as fillets and small-radius arcs, were suppressed during modeling. All bearing components (rollers, inner/outer rings) utilize bearing steel GCr15, with detailed mechanical properties provided in Table 2. The strain-transferring sensor brackets employ structural steel, with relevant material parameters listed in Table 3.

To select the optimal measurement scheme, simulation experiments were conducted using Ansys. The roller-to-raceway contacts were modeled as frictional contacts with a coefficient of 0.005, where the inner surface of the outer ring and outer surface of the inner ring were designated as target surfaces, while the tapered roller’s outer surface served as the contact surface. For the bracket-to-bore connection, the bore wall was set as the target surface and the bracket’s cylindrical side as the contact surface, also configured with frictional contact. The physical interference fit between the bracket and roller bore was simulated via offset settings in the analysis.

**Figure 14:**
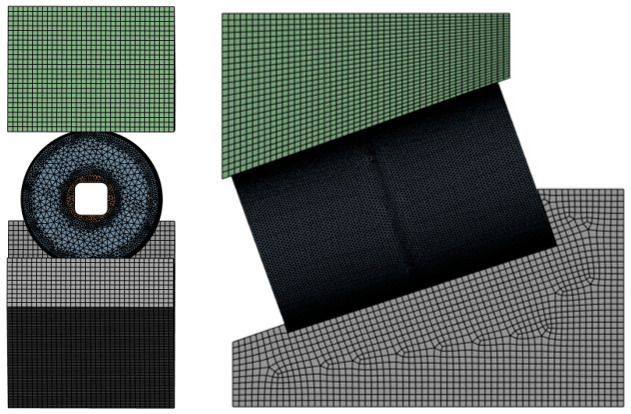
Meshing of the roller and bracket.

To ensure the reliability of numerical solutions, mesh independence verification was conducted prior to formal computation. By gradually increasing mesh density while maintaining accuracy, computational efficiency was maximized to establish the final mesh scheme. The inner/outer rings were meshed using the multizone method, with a maximum element size of 3 mm to control computational load. Rollers and brackets were discretized with tetrahedral elements: rollers at 3 mm global size, while brackets underwent refinement to 1.6 mm. Local mesh refinement was applied to roller outer surfaces and bore walls, yielding the final meshed model shown in Figure 14.

**Figure 15:**
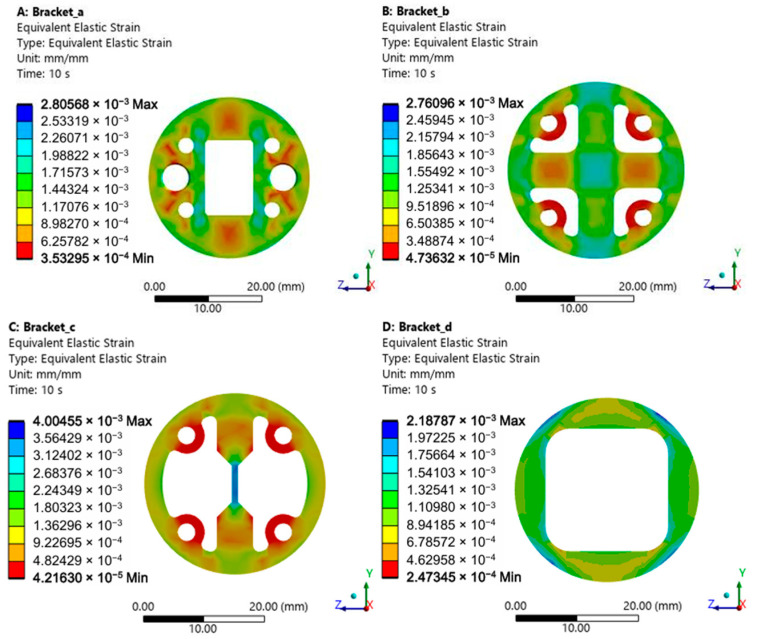
Equivalent strain distribution on the bracket: (**A**) Rectangular measuring bracket; (**B**) Cross-shaped bracket; (**C**) Single-beam bracket; (**D**) Annular bracket.

A fixed constraint was applied to the inner surface of the bearing outer ring, while remote displacement constraints were imposed on the rollers to restrict movement relative to the raceways. The remote displacement constraint applied to the roller restricts its translational degrees of freedom along the three coordinate axes relative to the inner/outer rings while permitting unrestricted rotational degrees of freedom about these axes. Loading was applied normal to the inner surface of the bearing inner ring. The analysis was divided into 10 steps, with roller loads incrementing from 50 to 500 kN. Computations were performed at 50 kN increments, with convergence tests conducted under these simulation conditions to guarantee the reliability of the results. The resulting equivalent strain on the bracket is shown in Figure 15, where the horizontal and vertical axes correspond to the global *Z*-axis and *Y*-axis, respectively.

**Figure 16:**
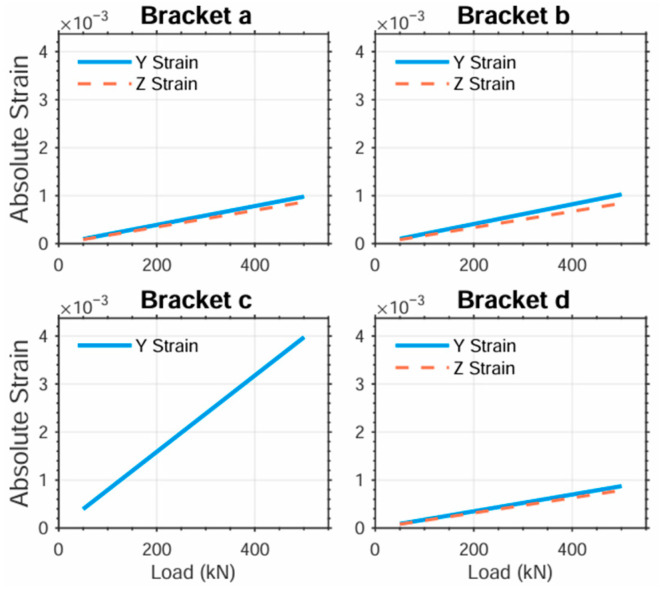
Regional strain on the bracket.

Fixed-size measurement zones were created at simulated strain gauge locations on the bracket surface. Strain values along the gauges’ sensitive directions were extracted within these zones. For consistent comparison across regions, absolute values of the extracted strains were adopted. Figure 16 presents the load-dependent strain variations across different zones for all four bracket designs.

The results demonstrate that the measurement scheme in Figure 10c exhibits significantly greater strain variation range than the others. Figure 10a,b,d show comparable load sensitivity, while Figure 10d yields the smallest strain variation.

Given the influence of actual bridge configurations during measurement, strain values extracted from different bridge schemes are processed to characterize strain variations via relative strain measurements. For the quarter-bridge (QB) configuration, the output voltage is:

(25) $$U_{QB}=\frac{\Delta R}{4R+2\Delta R}U_{e}$$

Under typical conditions where Δ*R* << *R*, the expression can be simplified to:

(26) $$U_{QB}=\frac{\Delta R}{4R}U_{e}$$

The output voltage *U*_QB_ of the bridge is proportional to the excitation voltage *U_e_*. With *U_e_* held constant, it exhibits a monotonic linear relationship with the relative resistance variation Δ*R*/*R* of the active bridge arm. Similarly, the output voltage for the half-bridge (HB) configuration is:

(27) $$U_{HB}=\frac{\Delta R}{2R}U_{e}$$

The output voltage for the full-bridge (FB) configuration is:

(28) $$U_{FB}=\frac{\Delta R}{R}U_{e}$$

By synthesizing Equations (26)–(28), the relationship among the output voltages of the three DC bridge configurations under identical strain-induced resistance variations in active arms is established as:

(29) $$U_{FB}=2U_{HB}=4U_{QB}$$

**Figure 17:**
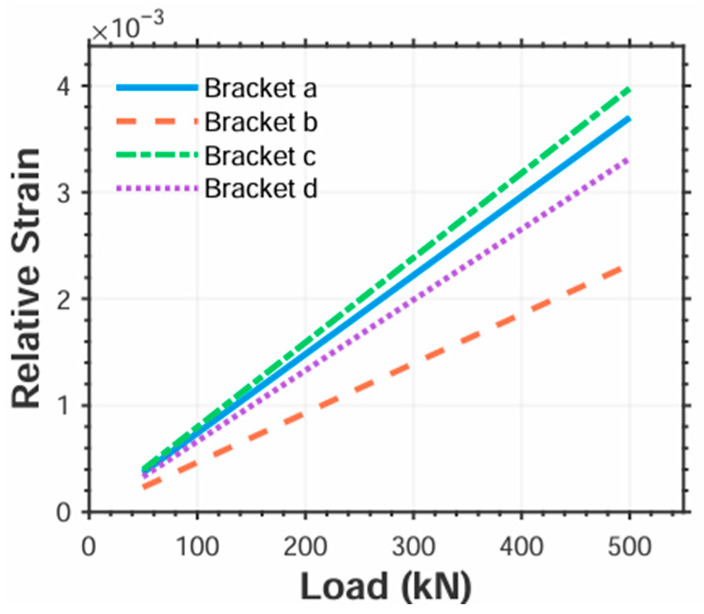
Regional relative strain versus load.

Based on this principle, the calibrated strain is calculated by multiplying the absolute strain by scaling factors: ×1 for quarter-bridge (QB), ×2 for half-bridge (HB), and ×4 for full-bridge (FB) configurations. The resulting relative strain variations versus load for each scheme are shown in Figure 17.

The results demonstrate that all four relative strain vs. load curves acquired under actual bridge configurations exhibit excellent linearity (Figure 17). Brackets c display substantially greater relative strain variation ranges, making this configuration preferable for strain acquisition under identical loading conditions.

Linear fitting of strain–load data for Bracket c yields:

(30) $${Ε}_{BRKTc}=7.95\times 10^{-6}q-9.33\times 10^{-9}$$

From Equations (23) and (30), the load–strain conversion coefficient for hollow tapered rollers is determined as *K_q_* = 1.26 × 10^5^. Contact loads calculated via *K_q_* and strain are summarized in Table 4.

## 4. Quantitative Strain–Phase–Load Correlation Method Incorporating Roller Rotation Phase

The correspondence between strain and contact load described earlier is considered when the strain reaches its maximum under a specific contact load, equivalent to the relationship at peak sensitivity of the measurement point. This analysis does not account for the dynamic characteristics of measurement sensitivity varying with the roller phase angle. However, during actual roller operation, its rotation causes continuous phase changes. Given the significant differences in load measurement sensitivity at different phase angles, the variation in measurement sensitivity due to phase angle changes must be considered when determining contact loads based on strain measurements.

### 4.1. Quantitative Strain–Phase–Load Correlation Method

**Figure 18:**
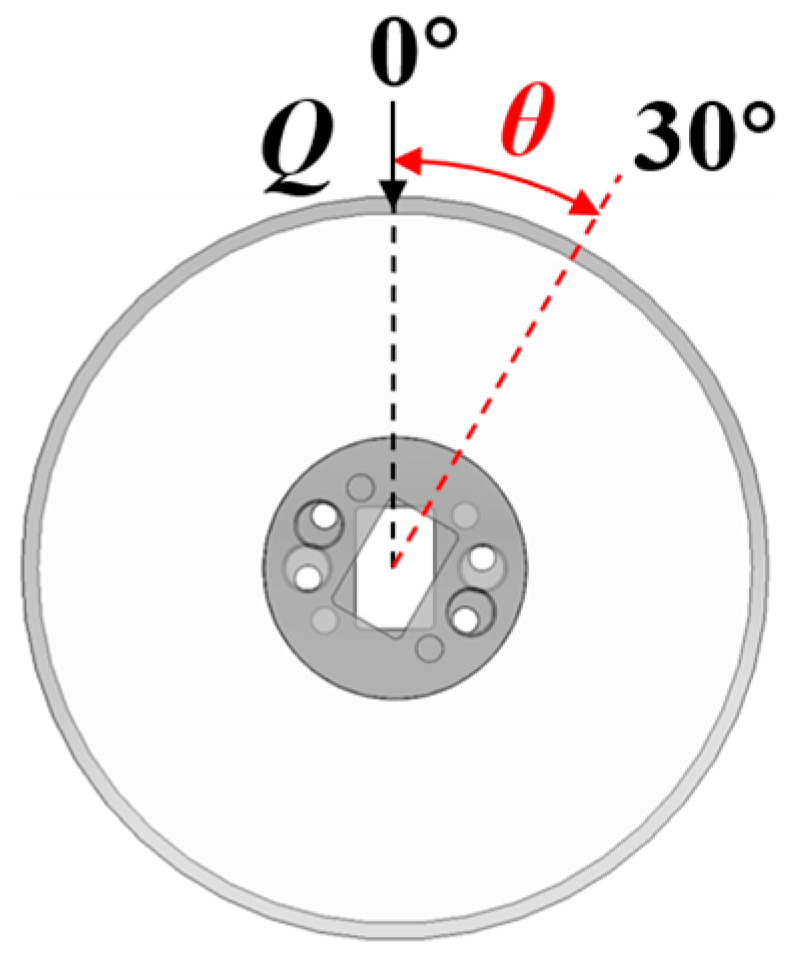
Measurement direction and angle.

Considering that the angle *θ* between the contact load direction and strain gauge measurement direction continuously varies during roller rotation (as shown in Figure 18), the measured strain value becomes variable under constant load.

**Figure 19:**
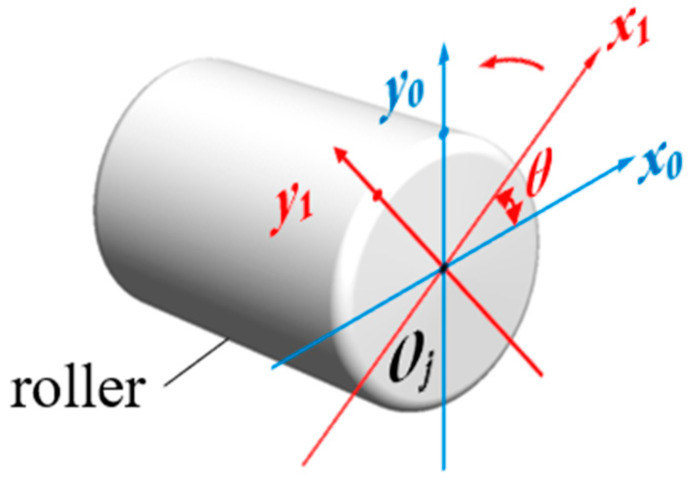
The roller coordinate system in a 2D plane.

To determine the relative positional relationship between the roller and fixed point, two coordinate systems are established with the roller center O*_j_* as the origin: the roller coordinate system *x*_0_*y*_0_ and the roller rotating coordinate system *x*_1_*y*_1_ (Figure 19). The rotation angle between these coordinate systems is denoted by *θ*.

**Figure 20:**
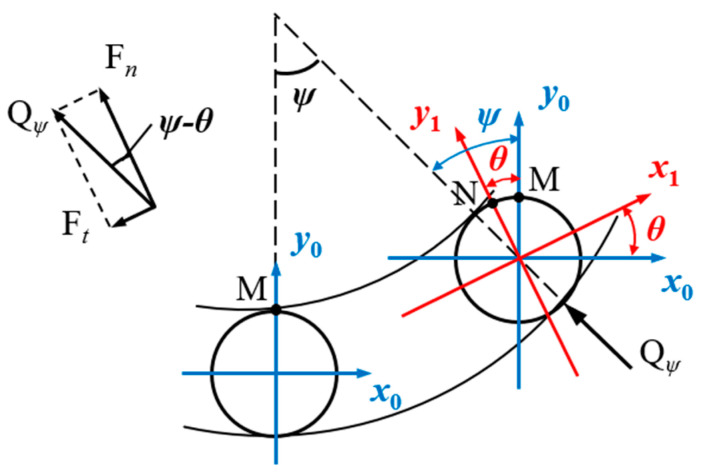
Positional relationship variation.

For simplified analysis, the measurement area on the roller inner wall is abstracted as a fixed point. Considering positional changes of the roller within the bearing (initially positioned directly below the bearing), the intersection point of the positive *y*_0_-axis with the roller edge is selected as the fixed point, designated as position M, where the applied load represents the roller contact load. As the roller rotates, it undergoes revolution angle *ψ* relative to the inner ring while simultaneously rotating about its own axis by angle *θ*, causing the fixed point to move from position M on the *y*_0_-axis to position N on the *y*_1_-axis as illustrated in Figure 20.

At position N, the tangential load at the fixed point is denoted as *F_t_,* and the normal load as *F_n_*. The contact load *Q_ψ_* on the roller is decomposed as follows:

(31) $$F_{n}=Q_{\psi }\cos\left(\psi -\theta \right)$$

(32) $$F_{t}=Q_{\psi }\sin\left(\psi -\theta \right)=Q_{\psi }\sin\varphi$$

According to geometric relationships, the tangential strain at the fixed point can be approximated as:

(33) ε=Acos2φ+B

By selecting strain values *ε* at *φ* = 0° and 90° under contact load *Q_ψ_* to solve the above equation, parameters *A* and *B* can be determined. The equation can then be rewritten as:

(34) $$\varepsilon =\frac{Q_{\psi }-b_{1}}{k_{1}}\cos2\varphi +B$$

Here, coefficient *A* characterizes the linear relationship between roller contact load and tangential strain at the fixed point. Parameters *k*_1_ and *b*_1_ are fitting coefficients, while *B* represents a systematic deviation correction term that requires experimental determination.

### 4.2. Validation Using Ansys-Based Simulation

**Figure 21:**
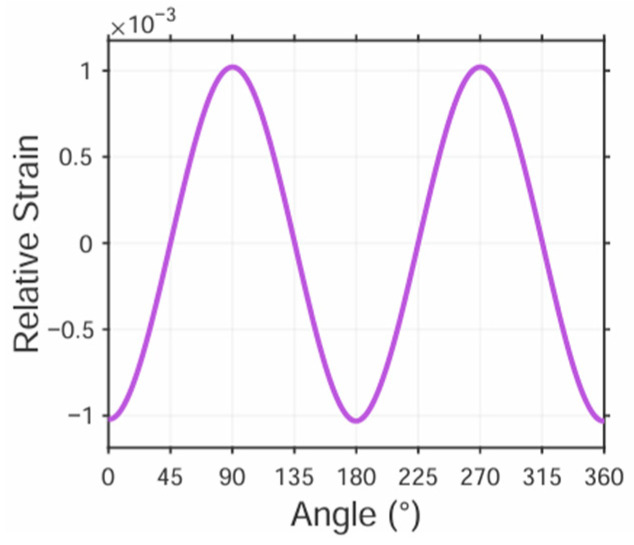
Regional relative strain versus angle.

To achieve the objective of indirectly determining contact loads via strain measurements, the acquired data must undergo processing and calculation. This necessitates that the curve depicting the relationship between measured strain values and roller phase angles should be readily analyzable. For this purpose, a 100 kN load is applied to the roller surface while varying the angle between the measurement area and load direction from 0° to 360° in 15° increments. At each angular position, strain values are recorded. The local coordinate system of the bracket is defined according to the sensitive direction of the strain gauges within the measurement area. Strain data extracted in this local coordinate system undergo spline interpolation, connecting all data points to generate the relative strain vs. angle curve, as shown in Figure 21.

The above figure demonstrates that the relative strain curves measured on Bracket (c) exhibit smooth variation with angle, closely approximating sinusoidal patterns. This characteristic significantly reduces data processing complexity.

**Figure 22:**
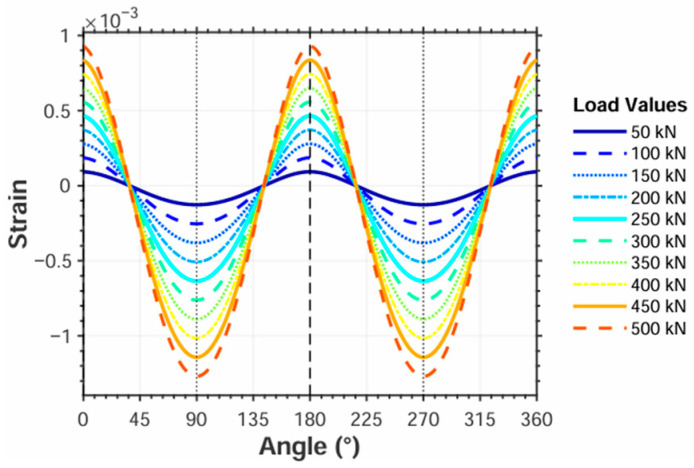
Strain versus angle under different loads.

To verify the contact load determination method for tapered hollow rollers, a finite element simulation was performed using Ansys. The analysis was conducted with 10 load increments, applying loads ranging from 50 to 500 kN to the roller. The angle between the load application position and strain measurement area (refer to Figure 21) was varied from 0° to 180° in 15° increments. Tangential strain values across the measurement area were recorded, yielding strain versus angle curves under varying loads, as shown in Figure 22.

For each load case, strain values at 0° and 90° are selected from Figure 22 to calculate the parameters in Equation (33), with detailed results presented in Table 5.

Based on the tabulated data, linear fitting of parameter *A* via the least squares method yields *k*_1_ = 4.5507 × 10^5^ and *b*_1_ = −0.0030, with a sum of squared errors (SSE) of 0.0003, confirming the result credibility. Similarly, fitting parameter *B* produces *k*_2_ = −2.9350 × 10^6^ and *b*_2_ = 0.0018 (SSE = 0.0004), also demonstrating reliability. Given that *b*_1_ and *b*_2_ are negligible compared to *k*_1_ and *k*_2_, we omit *b*_1_ and *b*_2_ in subsequent calculations. The roller contact load is, therefore, determined by Equation (34):

(35) $$Q_{\psi }=\frac{k_{1}k_{2}\varepsilon +b_{1}k_{2}\cos2\varphi +b_{2}k_{1}}{k_{1}+k_{2}\cos2\varphi }$$

(36) $$Q_{\psi }=\frac{k_{1}k_{2}\varepsilon }{k_{1}+k_{2}\cos2\varphi }$$

By substituting the simulated strain values at 5°, 10°, and 15° into Equation (36), the predicted contact loads are calculated. These values are then compared with the simulated contact loads, with comparative results presented in Table 6, Table 7 and Table 8, respectively.

The tabulated data demonstrate that the predicted contact loads are systematically lower than the simulated contact loads, with the prediction deviation exhibiting a progressive increase at larger load application angles.

## 5. Experimental Validation

To achieve the objective of installing sensors within the roller while preventing significant degradation in structural rigidity, the bore diameter should be minimized. Through a comprehensive assessment, the internal diameter is determined as 26 mm. Based on the computational model proposed earlier, the roller contact load can be derived from measured strain values, with dynamic variations in roller angular position incorporated to enable real-time load monitoring. This section details the calibration and testing of key model parameters, along with the architecture of the measurement circuitry of the testing system and its calibration experiments.

### 5.1. Design of Roller Contact Load Testing System

**Figure 23:**
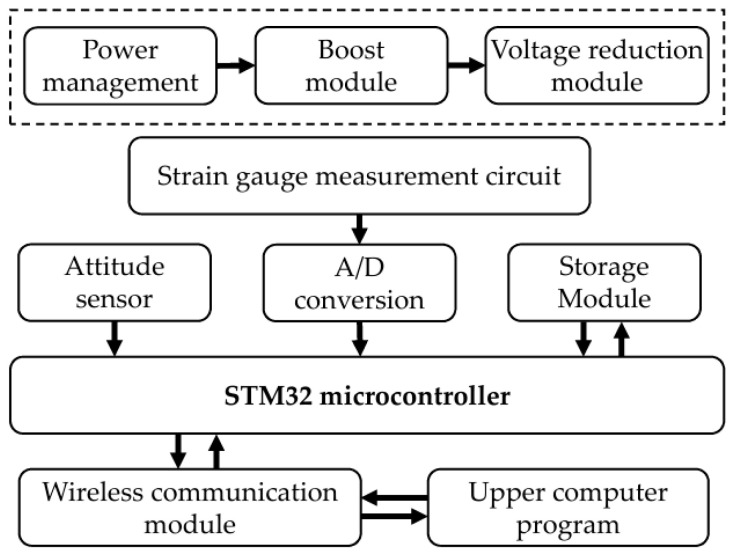
Circuit configuration of the roller contact load test system.

The measurement circuitry of the testing system is configured as shown in Figure 23. Strain gauges are mounted at the mid-section of the roller bore’s inner wall, oriented with their sensitive direction aligned circumferentially (tangential to the bore). The specific parameters of the strain gauges used in the experiment are detailed in Table 9. An attitude detection chip integrated onto the circuit board monitors the angular position of the roller. The entire measurement module is mechanically fixed within the bore, maintaining rotational synchronization with the roller.

### 5.2. Calibration Tests

**Figure 24:**
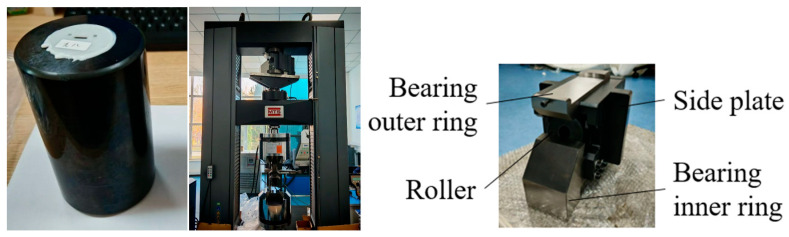
Calibration test equipment for the roller contact load test system.

The calibration tests require dedicated procedures to establish quantitative correspondence between strain and voltage signals, as strain gauges fundamentally output voltage variations. Primary equipment includes a hydraulic press, roller contact load testing system, and specialized roller loading fixtures (Figure 24), where the press applies controlled loads to simulate roller loading conditions. Through precise load regulation, diverse contact load scenarios are replicated. The test roller is positioned in a custom fixture that authentically simulates bearing loading states: its upper section mimics the bearing outer race rigidly connected to the press’s stationary crossbeam; the lower section replicates the inner race mounted on the mobile loading platen, while side restraints constrain lateral movement during loading to ensure force transmission along the predetermined direction.

The experimental setup utilized an E45.605 electromechanical universal testing machine (600 kN capacity) manufactured by MTS Systems Corporation. This system enables force-controlled, displacement-controlled, or strain-controlled testing within a 5 N to 600 kN loading range, with a positional resolution of 0.016 μm.

**Figure 25:**
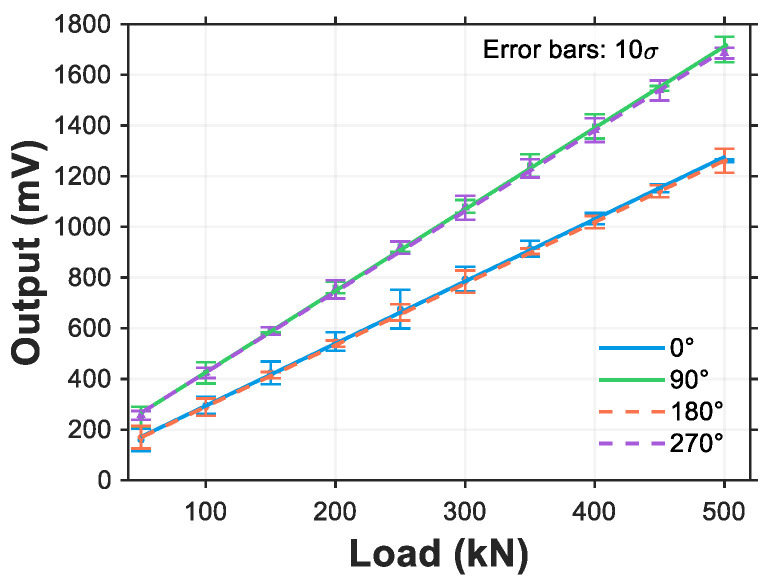
Calibration test: load versus output curve.

To validate the simulation results, loads ranging from 0 to 500 kN were applied to the roller in 50 kN increments, with strain gauge readings recorded at each step. Additionally, to verify the patterns at different angles, measurements were conducted at roller angles of 0°, 90°, 180°, and 270°. Three repeated measurements were performed at each angle, yielding the experimental results shown in Figure 25.

As shown in the figure, a strong linear relationship exists between strain gauge readings and applied loads. This linear pattern is identical at 0° and 270° while being consistent at 90° and 180°. The relationship between load and output value can be expressed as:

(37) Q=kA+b

where *Q* represents the applied load, *A* denotes the strain gauge output value, and *b* indicates the sensor’s zero offset. Linear fitting was performed between the strain gauge outputs and applied loads, yielding the following results: at 0° and 270° positions, the fitted equation is *Q* = 0.3858*A* − 11.7143 with a coefficient of determination *R*^2^ = 0.9991, where *k*_1_ = 0.3858 and *b*_1_ = −11.7143; at 90° and 180° positions, the fitted equation is *Q* = −0.31*A* − 37.90 with *R*^2^ = 0.9995, demonstrating excellent correlation between strain measurements and applied loads.

**Figure 26:**
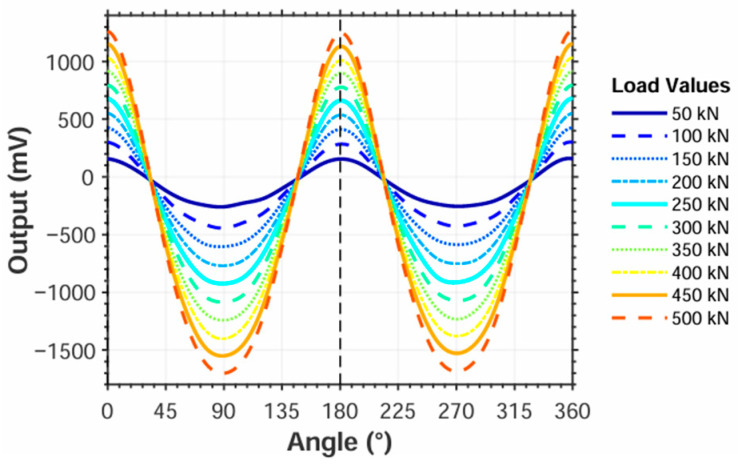
Calibration test: angle versus output curve.

To investigate the relationship between rotational angle and output values, the roller was rotated from 0° to 360° in 15° increments while applying 0 to 500 kN loads at 50 kN intervals per angular position, with the resulting variation curves between roller angle and output values, as shown in Figure 26.

Comparison with Figure 22 confirms that the experimentally measured relationship between strain gauge output and rotation angle aligns with simulation results. The strain output values at 0° and 90° were used to calculate parameter *B*. Linear fitting of parameter *B* versus applied load yielded the equation *Q* = −2.4952*B* − 27.6555 with a coefficient of determination *R*^2^ = 0.9989, where *k*_2_ = −2.4952 and *b*_2_ = −27.6555, indicating excellent linear correlation. The fitted values were substituted into Equation (35) to determine roller contact loads based on angular position and strain output. Subsequently, measurement errors relative to actual loads were calculated using Equation (38), with results presented in Table 10, Table 11, Table 12 and Table 13.

(38) $$\delta =\frac{Q_{a}-Q_{m}}{Q_{a}}$$

where *Q_a_* represents the actual load applied to the roller, while *Q_m_* denotes the measured contact load of the roller.

The above data demonstrate that the proposed strain-based contact load calculation method for rollers effectively predicts roller contact loads from strain measurements, with errors confined within 5%.

## 6. Conclusions

This study focuses on large tapered roller bearings for wind turbines and proposes an innovative method for acquiring contact loads through strain measurements within roller bores. The principal conclusions are summarized as follows:

1. The influence of sensor placement and bridge configurations was analyzed to optimize the measurement scheme. Systematic investigation determined that the optimal solution involves mounting strain gauges on internal brackets, effectively capturing strain signals while maximizing structural rigidity of bored rollers.
2. Load–strain mapping principles were established and experimentally validated. Finite element simulations systematically revealed strain gauge responses under varying load magnitudes and phase angles, with subsequent experiments confirming the accuracy of these patterns.
3. A contact load calculation methodology was developed and the accuracy was verified. A strain signal inversion-based computational approach was proposed, with key parameters calibrated through dedicated roller tests. Application at specific phase angles demonstrated effective contact load acquisition with measurement accuracy exceeding 95%.

The proposed method currently enables accurate measurement of roller contact loads. However, rotational motion during roller operation induces phase-dependent variations in strain measurement sensitivity. This results in marginally reduced measurement accuracy at specific angular positions, though overall accuracy remains relatively stable. To achieve higher precision, further investigation into strain sensitivity fluctuations is warranted. Additionally, the influence mechanisms of complex operating conditions—particularly non-uniform loading scenarios—on contact load measurement require deeper exploration to enhance the method’s robustness in real-world operational environments.

## Figures and Tables

**Table 1:** Main structural parameters of a tapered roller bearing.

Parameter	Value
Number of Rollers	41
Roller–Outer Ring Contact Angle	20°
Roller–Inner Ring Contact Angle	18°27′
Large-End Diameter of Roller	85 mm
Effective Contact Length of Roller	129 mm
Bearing Pitch Diameter	2063 mm

**Table 2:** Material parameters of GCr15.

Parameter	Unit	Value
Density	kg/m^3^	7830
Poisson’s Ratio	-	0.3
Young’s Modulus	MPa	2.19 × 10^5^

**Table 3:** Material parameters of structural steel.

Parameter	Unit	Value
Density	kg/m^3^	7850
Poisson’s Ratio	-	0.3
Young’s Modulus	MPa	2.0 × 10^5^

**Table 4:** Strain values versus contact load.

Strain	Contact Load/kN
3.9741 × 10^−4^	50.07
7.9482 × 10^−4^	100.15
1.1922 × 10^−3^	150.22
1.5896 × 10^−3^	200.29
1.9871 × 10^−3^	250.37
2.3845 × 10^−3^	300.44
2.7819 × 10^−3^	350.52
3.1793 × 10^−3^	400.59
3.5767 × 10^−3^	450.66
3.9741 × 10^−3^	500.74

**Table 5:** Calculated parameters A and B for a hollow tapered roller.

Q_φ_/kN	A (×10^4^)	B (×10^4^)
50	1.0987	−0.1704
100	2.1975	−0.3407
150	3.2962	−0.5111
200	4.3950	−0.6814
250	5.4937	−0.8518
300	6.5925	−1.0221
350	7.6912	−1.1925
400	8.7900	−1.3628
450	9.8887	−1.5332
500	10.9875	−1.7036

**Table 6:** Simulated vs. predicted contact loads of a hollow tapered roller (*φ* = 5°).

Simulated Value/kN	Predicted Value/kN	Deviation/kN	Error/%
50	49.66	0.34	0.689
100	99.31	0.69	0.689
150	148.97	1.03	0.689
200	198.62	1.38	0.689
250	248.28	1.72	0.689
300	297.93	2.07	0.689
350	347.59	2.41	0.689
400	397.24	2.76	0.689
450	446.90	3.10	0.689
500	496.55	3.45	0.689

**Table 7:** Simulated vs. predicted contact loads of a hollow tapered roller (*φ* = 10°).

Simulated Value/kN	Predicted Value/kN	Deviation/kN	Error/%
50	48.45	1.55	3.090
100	96.91	3.09	3.090
150	145.36	4.64	3.090
200	193.82	6.18	3.090
250	242.27	7.73	3.090
300	290.73	9.27	3.090
350	339.18	10.82	3.090
400	387.64	12.36	3.090
450	436.09	13.91	3.090
500	484.55	15.45	3.090

**Table 8:** Simulated vs. predicted contact loads of a hollow tapered roller (*φ* = 15°).

Simulated Value/kN	Predicted Value/kN	Deviation/kN	Error/%
50	46.58	3.42	6.836
100	93.16	6.84	6.836
150	139.75	10.25	6.836
200	186.33	13.67	6.836
250	232.91	17.09	6.836
300	279.49	20.51	6.836
350	326.07	23.93	6.836
400	372.66	27.34	6.836
450	419.24	30.76	6.836
500	465.82	34.18	6.836

**Table 9:** Strain gauge specifications.

Parameter	Value
Type	BE120-3AA-P2K
Ohms	120.0 ± 0.1
Gage Factor	2.22 ± 1%
Active Grid Size	2.8 × 2.0 mm

**Table 10:** Calibration test: actual vs. measured loads (at 0°).

Actual Value/kN	Measured Value/kN	Error/%
49.97	47.8918	4.16
102.63	100.2516	2.32
151.11	149.9024	0.80
202.07	200.4936	0.78
252.68	253.4219	−0.29
300.92	300.4914	0.14
351.14	351.3049	−0.05
400.02	400.4828	−0.12
451.43	451.1876	0.05
499.97	495.9722	0.80

**Table 11:** Calibration test: actual vs. measured loads (at 90°).

Actual Value/kN	Measured Value/kN	Error/%
54.54	51.8643	4.91
104.91	101.0607	3.67
152.05	148.9871	2.01
202.52	200.6425	0.93
250.15	249.6089	0.22
300.53	300.5040	0.01
350.57	350.9279	−0.10
402.17	402.4420	−0.07
450.25	450.0172	0.05
501.89	497.3399	0.91

**Table 12:** Calibration test: actual vs. measured loads (at 180°).

Actual Value/kN	Measured Value/kN	Error/%
49.99	47.8748	4.23
99.73	96.3589	3.38
150.88	146.4544	2.93
201.32	196.2641	2.51
252.53	246.2964	2.47
301.92	295.7445	2.05
353.63	348.2109	1.53
400.13	395.0541	1.27
451.43	447.4469	0.88
503.12	497.1960	1.18

**Table 13:** Calibration test: actual vs. measured loads (at 270°).

Actual Value/kN	Measured Value/kN	Error/%
52.87	50.6448	4.21
102.82	99.3945	3.33
149.81	145.0797	3.16
199.81	193.8589	2.98
250.43	245.6553	1.91
300.32	295.7064	1.54
352.19	347.7597	1.26
400.18	395.1192	1.26
449.52	443.5538	1.33
500.43	494.9456	1.10

## Data Availability

Data are contained within the article.
